# Supplementary material for: Drug prescribing and dispensing practices in regional and national referral hospitals of Eritrea: Evaluation with WHO/INRUD core drug use indicators
Source: PLoS One. 2022 Aug 19;17(8):e0272936. doi: 10.1371/journal.pone.0272936 (PMC9390936; doi:10.1371/journal.pone.0272936)
Supplement: S1 File — (PDF) [file pone.0272936.s001.pdf]

Prescribing indicator recording form

Location: ____

Investigator: ______________________________________ **Date:** _______________

Seq. #	Type	Date prescription was written	Age (yrs.)	# of Drugs prescribed	# of Generics	Presence of Antibiotic (0/1)*	Presence of Injection (0/1)*	# of drugs on ENLM	Diagnosis
1.	Retrospective								
2.									
3.									
4.									
5.									
6.									
7.									
8.									
9.									
10.									
11.									
12.									
13.									
14.									
15.									
16.									
17.									
18.									
19.									
20.									
21.									
22.									
23.									
24.									
25.									
26.									
27.									
28.									
29.									
30.									
31.									
32.									
33.									
34.									
35.									
Sub total									

*** 0 = No   *1= Yes**

Detailed Prescribing Encounter Form

Location: ____________________________________

Investigator: ___________________________________ **Date:** ____________________

<table>
<tr><th>ID #</th><th>Date</th><th>Name</th><th>Age</th><th>Sex</th><th colspan="2">Prescriber</th></tr>
<tr><td></td><td></td><td></td><td></td><td></td><td colspan="2"></td></tr>
<tr><td rowspan="4">Health Problems</td><td colspan="4">Health Problem Description (Chief compliant)</td><td colspan="2" rowspan="4"></td></tr>
<tr><td colspan="4">1.</td></tr>
<tr><td colspan="4">2.</td></tr>
<tr><td colspan="4">3.</td></tr>
<tr><td>Drugs</td><td colspan="2">Name and Strength</td><td colspan="2">Quantity</td><td>Unit price</td><td>Total price</td></tr>
<tr><td></td><td colspan="2">1.</td><td colspan="2"></td><td></td><td></td></tr>
<tr><td></td><td colspan="2">2.</td><td colspan="2"></td><td></td><td></td></tr>
<tr><td></td><td colspan="2">3.</td><td colspan="2"></td><td></td><td></td></tr>
<tr><td></td><td colspan="2">4.</td><td colspan="2"></td><td></td><td></td></tr>
<tr><td></td><td colspan="2">5.</td><td colspan="2"></td><td></td><td></td></tr>
<tr><td colspan="6">Grand total price</td><td></td></tr>
</table>
