# Supplementary material for: Drug prescribing and dispensing practices in regional and national referral hospitals of Eritrea: Evaluation with WHO/INRUD core drug use indicators
Source: PLoS One. 2022 Aug 19;17(8):e0272936. doi: 10.1371/journal.pone.0272936 (PMC9390936; doi:10.1371/journal.pone.0272936)
Supplement: S2 File — (PDF) [file pone.0272936.s002.pdf]

Patient indicator recording form

Location: ____

Investigator: ____ **Date:** _____________

Seq. #	Patient identifier (Sex)	Consulting Time (mins)	Dispensing Time (secs)	# of Drugs Prescribed	# of Drugs actually Dispensed	# of drugs Adequately Labelled	Patient Knows Correct Dosages (0/1)*
1							
2							
3							
4							
5							
6							
7							
8							
9							
10							
11							
12							
13							
14							
15							
16							
17							
18							
19							
20							
21							
22							
23							
24							
25							
26							
27							
28							
29							
30							
**Total**							
**Average**							
**Percentage**					_____ % of prescribed	_____ % of dispensed	_____ % of pts. know correct dosage

* 0= No 1= Yes
