# Supplementary material for: Drug prescribing and dispensing practices in regional and national referral hospitals of Eritrea: Evaluation with WHO/INRUD core drug use indicators
Source: PLoS One. 2022 Aug 19;17(8):e0272936. doi: 10.1371/journal.pone.0272936 (PMC9390936; doi:10.1371/journal.pone.0272936)
Supplement: S3 File — (PDF) [file pone.0272936.s003.pdf]

<u>Facility Indicator Recording Form</u>

Location: ____
Investigator: ____ **Date: _____________**

**Facility indicators**	**Results**
Availability of Eritrean National Drug List (Yes/No)	
Availability of Eritrean National Formulary (Yes/No)	
Availability of Eritrean Treatment Guidelines (Yes/No)	
Percentage availability of key indicator drugs (%)	

COMMENTS:

____
____
____
____
____
____

SIGNATURE: ______________
