# Supplementary material for: Drug prescribing and dispensing practices in regional and national referral hospitals of Eritrea: Evaluation with WHO/INRUD core drug use indicators
Source: PLoS One. 2022 Aug 19;17(8):e0272936. doi: 10.1371/journal.pone.0272936 (PMC9390936; doi:10.1371/journal.pone.0272936)
Supplement: Attachment — Submitted filename: Response to Reviewers.docx [file pone.0272936.s004.docx]

**Author response to Reviewer’s comments**

We would like to thank our academic editor and reviewers for their invaluable inputs and constructive comments that are helpful to massively improve the quality of the manuscript. After careful consideration of the points raised by academic editor and reviewers, a point-by-point response are as follows:

**Academic editor**

1. How was non-polypharmacy defined in the study? It is mentioned that two or less were defined as non-polypharmacy. Usually polypharmacy is defined as five or more drugs in a prescription. “*Index of non-polypharmacy was measured by dividing average number of drugs by 1.8”.* Why 1.8? And on page 7, line 136 the formula mentions observed value in the denominator.

**Response:** It is well noted. The definition of polypharmacy in the manuscript was inappropriate and therefore omitted from the text. Besides, the statement “*Index of non-polypharmacy was measured by dividing average number of drugs by 1.8”* was a typing error and therefore removed from the text. Furthermore, in the result section of the study the concept of “*Index of non-polypharmacy was measured by dividing the optimal value (taken as 1.8) by the average number of drugs”*. The index of non-polypharmacy was expressed in terms of the average number of drugs per prescription.

2. What is the source of WHO standard as mentioned in the tables? The term optimal value is also used at several places in the text. What is the difference between- WHO standard and optimal values? It is worthwhile to give a table mentioning the optimal values in the study and their references.

**Response:** The reference for the optimal values was cited. There is no difference between the WHO standard and optimal values (used interchangeably). Moreover, an additional table (1) that mentions the WHO standard (optimal values) for all the three indicators alongside their references is added in the method section.

3. The naming of healthcare facilities needs to be uniform in the manuscript especially tables.

**Response:** Accepted

4. The methods section doesn’t mention about the country performance indicators. How was the value of 1 calculated for indices of non-polypharmacy and rational injection use?

**Response:** The country performance indicators for indices of non-polypharmacy and rational injection use was calculated by the concept (optimal value/observed value). The optimal value for non-polypharmacy was taken as 1.8, whereas the optimal value for rational injection use was taken as 24.1. Moreover, the observed value was taken as the overall (average) of the hospitals in each item within the indicators. Optimal index that yielded more than one was taken as one as the maximum value of the optimal index is one. The above-mentioned calculation approach for the country performance indicators was included in the ‘method’ section within the revised manuscript.

5. The implications of the study findings need to be discussed in greater details for all stakeholders especially policy makers.

**Response:** Accepted. The implications of the study findings are discussed in greater details for all stakeholders. The discussion section is modified in the revised manuscript.

6. There are some language errors and some discrepancies in the numbers mentioned in the text and tables.

**Response:** Accepted. A massive editing is made on the manuscript. We have made 320 insertions, 236 deletions, 2 moves and 13 formatting in the revised section. Accordingly, the reference section is fully revised. Please refer the ‘revised manuscript with track changes’.

**Reviewer 1**

**-**Statistical analysis: kindly develop adjusted result

**Response:** Adjusted result could not be obtained as there is only one independent variable namely, hospital. All the other variable such as average number of medicines per prescription, average consultation and dispensing time and so on are assessed on their potential difference across the hospitals.

-Ethical approval sound appropriate. Please detail ethical approval body with address. Add Reference number of approval letter.

**Response:** Accepted

-Please separate conclusion and recommendation.

**Response:** Accepted.

-Your key message in a box.

**Response:** The key message is already included in the separated recommendation section.

-Please try to use recent references add or replace more than 5 years old.

**Response:** This is a very important comment. Our paper has been prepared before 2 years. Hence, your comment is valuable and we included recent available studies in the introduction and discussion sections, while removing too old references. Besides, for WHO and other guidelines that were prepared a long time ago and which were used as a source material for the methodology we preferred to keep them in the references.

**Reviewer 2**

1. Correction of grammar, spellings and punctuations to improve readability.

**Response:** Accepted. A massive editing is made on the manuscript. We have made 320 insertions, 236 deletions, 2 moves and 13 formatting in the revised section. Accordingly, the reference section is fully revised. Please refer the ‘revised manuscript with track changes’.

2. I notice that the term drug and medicine are used interchangeably, suggest sticking to the term medicine. Of course you will have to use Drug when referring to INRUD. But in other place make it uniform.

**Response:** Accepted. Where appropriate, the term ‘drug’ is replaced with the term ‘medicine’.

**Abstract**

1. Were the prescriptions collected from in-patients or out-patients or both? It’s not clear in the abstract.

**Response:** It was from out-patients.

2. Which disciplines were included? Coz prescribing patterns will be different. Was it all? Medicine, Surgery, Gyn & Obs, Paediatrics, etc. Again not clear in the abstract.

**Response:** All medical specialties (disciplines) were included.

3. In abstract SD is not reported for some parameters.

**Response:** Accepted. Standard deviation is reported for all parameters expressed as M (SD).

4. Since most of these are likely to be skewed by outlier values, its best to report median and range as well.

**Response:** Accepted. We reported it as mean for ease of comparison of the values with that of the WHO standard. Besides, at times median value is important we reported it. For instance, average consultation and dispensing time.

**Introduction**

1. Would benefit from a description regarding current practices in the country and a small introduction about its healthcare system (public vs. private, etc, etc)

**Response:** Accepted. The current practices in the country is included in the introduction. Moreover, a small introduction about the Eritrean healthcare system is given in the method section (study design and setting sub-section).

**Methods**

1. How big are these hospitals? An indicator about bed strength or an appropriate reference will be helpful for the reader.

**Response:** All the study sites (hospitals) are one of the biggest hospitals in the country. Orotta and Halibet hospitals are national referral hospitals that serves patients from all around the country. They are situated in the capital city of Eritrea (Asmara) and considered as a tertiary level services that provides lots of services such as out-patient, in-patient, maternity, paediatrics, and surgery and so on. Furthermore, the rest six hospitals are regional referral hospitals that serves patients referred or self-referred within the region they locate. They are considered as secondary level of service and are bigger in nature.

2. I now see that this is mainly OPD prescriptions. However, is it all specialties?

**Response:** Yes, it was from all specialties.

3. How many data collectors were involved?

**Response:** A total of 20 data collectors were involved in the study.

**Results**

1. As mentioned above mean may not be the best way to report. At least mention median and range within brackets.

**Response:** Accepted. At times Md (IQR) is important we reported it in some parameters like average number of medicines per prescription, average consultation and dispensing time.

**Discussion**

1. The implications of the study findings are not sufficiently discussed with appropriate references. For example, use of antibiotics.

**Response:** Accepted. The implications of the study findings are expressed in detail in the revised manuscript.

2. What is the impact of having a mixed group of patients from different specialties?

**Response:** It helps to get a complete picture of the rational use of medicines within a hospital.

3. Limitations of the study not described adequately.

**Response:** Accepted. We explored the possible limitations we have in the study and added in the revised manuscript.

Kind regards

Nuru Abdu (BPharm)

On behalf of the authors
