# Supplementary material for: Drug prescribing and dispensing practices in regional and national referral hospitals of Eritrea: Evaluation with WHO/INRUD core drug use indicators
Source: PLoS One. 2022 Aug 19;17(8):e0272936. doi: 10.1371/journal.pone.0272936 (PMC9390936; doi:10.1371/journal.pone.0272936)
Supplement: Attachment — Submitted filename: Response to Reviewers.docx [file pone.0272936.s005.docx]

**Author response to editor’s and reviewer’s comments**

We would like to thank our academic editor and reviewers for their invaluable inputs and constructive comments that are helpful to massively improve the quality of the manuscript. After careful consideration of the points raised by academic editor and reviewers, a point-by-point response are as follows:

**Academic editor**

1. The tables should include SD wherever mean is mentioned. Either of them (Mean SD/Median IQR) be reported for each variable depending upon distribution of data values.

**Response:** Thank you for this comment. SD is included wherever mean is mentioned. Besides, the authors preferred to report both the mean (SD) and median (IQR). Mean (SD) is used for ease of comparison with the WHO standard values and median (IQR) is included as some variables have outliers.

2. Table 1 has typo. Percent of drugs adequately labelled!

**Response:** Thank you for pointing out this issue. The typos error in table 1 is corrected in the revised manuscript.

3. There is discrepancy in the average number of drugs per encounter mentioned in the abstract, main results, discussion and the table 2. Needs to be rechecked.

**Response:** Comment is addressed in the revised manuscript.

4. Several statements in the discussion lack appropriate references viz. irrational and overuse of antibiotics……., injectable preparations………..

**Response:** Thank you for the great comment. The discussion section is revised and where necessary citations are added. Besides, some statements used as implications are the authors’ own ideas.

5. Limitations need to include implications of having a mixed group of patients from various specialties.

**Response:** Thank you for this comment. The study sites are regional and national referral hospitals having various specialties (disciplines). Thus, the study involved a mixed group of patients. So, the authors do not see the importance of adding it in the limitation section.

6. Discussion needs to be rechecked for consistency and appropriate references.

**Response:** Comment well noted. Necessary changes are made in the revised manuscript.

**Reviewer 1**

-Accept. As comments are addressed, I would like to recommend they should be accepted and published in PLOS ONE.

**Response:** Thank you for your review.

**Reviewer 2**

-Introduction: Line 72 must be ‘ensuring’

**Response:** Thank you for your comment. It was corrected in the revised manuscript.

**-**Methods: Line 80- ‘September 1 to January 31, 2018’ year for September to be mentioned.

**Response:** Comment well taken. Addressed in the revised manuscript.

-Tables 2, 5: The abbreviations M, Md, IQR to be expanded in foot note.

**Response:** Comment well taken. Addressed in the revised manuscript.

-Column headings could be made appropriate for all the values described in table. Values in Table 4 represent time, but the column heading does not mention time.

**Response:** Thank you for your suggestion. For ease of understanding, the authors added ‘minute’ and ‘second’ alongside the values for the consultation and dispensing time, respectively.

-Many are incomplete, example – Ref 1 is incomplete. Web pages are without a URL.

**Response:** Thank you for pointing out this issue. References are updated in the revised version.

Kind regards

Nuru Abdu (BPharm)

On behalf of the authors
